# Genetic Evidence for a Tacaribe Serocomplex Virus, Mexico

**DOI:** 10.3201/eid1606.091648

**Published:** 2010-06

**Authors:** Catherine C. Inizan, Maria N. B. Cajimat, Mary Louise Milazzo, Artemio Barragán-Gomez, Robert D. Bradley, Charles F. Fulhorst

**Affiliations:** University of Texas Medical Branch, Galveston, Texas, USA (C.C. Inizan, M.N.B. Cajimat, M.L. Milazzo, C.F. Fulhorst); Universidad Autónoma de Nuevo León, San Nicolas de los Garza, México (A. Barragán-Gomez); Texas Tech University, Lubbock, Texas, USA (R.D. Bradley)

**Keywords:** Viruses, Arenaviridae, zoonoses, Tacaribe virus, rodents, Mexico, dispatch

## Abstract

We isolated arenavirus RNA from white-toothed woodrats (*Neotoma leucodon*) captured in a region of Mexico in which woodrats are food for humans. Analyses of nucleotide and amino acid sequence data indicated that the woodrats were infected with a novel Tacaribe serocomplex virus, proposed name Real de Catorce virus.

The Tacaribe serocomplex (family *Arenaviridae,* genus *Arenavirus*) comprises 7 North American viruses (Table 1) and 15 South American viruses (*9*). The South American viruses include Guanarito virus, 4 other agents of hemorrhagic fever, and Pirital virus. Members of the rodent family *Cricetidae* are the principal hosts of the Tacaribe serocomplex viruses for which natural host relationships have been well characterized. For example, the southern plains woodrat (*Neotoma micropus*) in southern Texas is the principal host of Catarina virus (*4*), and the hispid cotton rat (*Sigmodon hispidus*) in southern Florida is the principal host of Tamiami virus (*6*).

**Table 1:** Natural hosts and geographic distribution of the North American Tacaribe serocomplex viruses

Virus*	Natural host(s)	State
Bear Canyon	Large-eared woodrat (*Neotoma macrotis*), California mouse (*Peromyscus californicus*)	California (*1*,*2*)
Big Brushy Tank	White-throated woodrat (*N. albigula*)	Arizona (*3*)
Catarina	Southern plains woodrat (*N. micropus*)	Texas (*4*)
Skinner Tank	Mexican woodrat (*N. mexicana*)	Arizona (*5*)
Tamiami	Hispid cotton rat (*Sigmodon hispidus*)	Florida (*6*)
Tonto Creek	White-throated woodrat (*N. albigula*)	Arizona (*3*)
Whitewater Arroyo	White-throated woodrat (*N. albigula*)	New Mexico (*7*)

*Arenaviruses antigenically and phylogenetically related to Whitewater Arroyo virus have been isolated from Mexican woodrats (*N. mexicana*) captured in New Mexico, a Mexican woodrat and bushy-tailed woodrat (*N. cinerea*) captured in Utah, and woodrats (*Neotoma* spp.) captured in Oklahoma (*8*).

A recent study found antibody to a Tacaribe serocomplex virus in white-toothed woodrats (*N. leucodon*) captured in Mexico (M.L. Milazzo, unpub. data). We report the determination of the nucleotide sequence of a 3352-nt fragment of the small (S) genomic segment of arenavirus AV H0030026 from RNA isolated from a white-toothed woodrat captured in 2005 in northeastern Mexico.

## The Study

**Figure 1:**
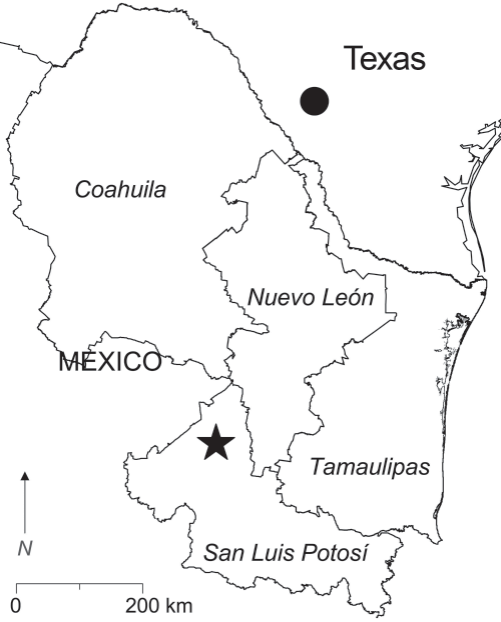
Southern Texas and 4 states in northeastern Mexico. The filled circle in southern Texas indicates the locality in which Catarina virus is enzootic. The star in San Luis Potosí indicates the location of the study site (23°49′5′′N, 100°49′54′′W). Antibody (immunoglobulin G) to Whitewater Arroyo virus previously was found in white-toothed woodrats (*Neotoma leucodon*), a Mexican woodrat (*N. mexicana*), and deer mice (*Peromyscus* spp.) captured in Nuevo León; white-throated woodrats (*N. albigula*) and white-toothed woodrats captured in San Luis Potosí; and deer mice (*Peromyscus* spp.) and a southern plains woodrat (*N. micropus*) captured in Tamaulipas (M.L. Milazzo, unpub. data).

Rodents were captured in a rural area 22.8 km north of the town of Real de Catorce in the municipality of Catorce, San Luis Potosí, Mexico (Figure 1). A total of 400 live-capture traps were set each night on 2 consecutive nights in the first week of August 2005. Each rodent caught was assigned a unique identification number. Samples of whole blood, samples of kidney and other solid tissues, and the skins and skeletons of the rodents were deposited into the Museum of Texas Tech University.

The blood samples were tested by ELISA (*10*) for antibody (immunoglobulin [Ig] G) to Whitewater Arroyo virus strain AV 9310135 (*7*). Samples of spleen and kidney from white-toothed woodrats TK133448 and TK133451, 7 other white-toothed woodrats, 2 antibody-positive Nelson’s pocket mice (*Chaetodipus nelsoni*), and an antibody-positive Merriam’s kangaroo rat (*Dipodomys merriami*) were tested for arenavirus by cultivation in monolayers of Vero E6 cells (*11*). Samples of kidney from the antibody-positive rodents were tested for arenavirus RNA by using heminested PCR assays. The first-strand cDNA for the PCR was synthesized by using SuperScript II Reverse Transcriptase (Invitrogen Life Technologies, Inc., Carlsbad, CA, USA) in conjunction with oligont 19C-cons (*2*). The nucleotide sequence alignments were analyzed by using MRBAYES 3.1.2 (*12*) in the computer software package PAUP*, version 4.0b10 (http://paup.csit.fsu.edu). (Details of the assays for IgG, arenavirus, and arenavirus RNA and the analyses of the amino acid sequence data and nucleotide sequence data are available from C.F.F.)

Nine white-toothed woodrats, 3 northern grasshopper mice (*Onychomys leucogaster*), 19 deer mice (*Peromyscus* spp.), 11 harvest mice (*Reithrodontomys* spp.), 13 Nelson’s pocket mice, 45 Merriam’s kangaroo rats, and 16 other kangaroo rats (*Dipodomys* spp.) were captured during 800 trap-nights; overall trap success rate was 116/800 (14.5%). IgG against Whitewater Arroyo virus was found in 2 (22.2%) of the white-toothed woodrats, 2 (15.4%) of the Nelson’s pocket mice, 1 (2.2%) of the Merriam’s kangaroo rats, and none of the 49 other rodents. Arenavirus was isolated from none of the 12 animals included in the virus assays.

**Figure 2:**
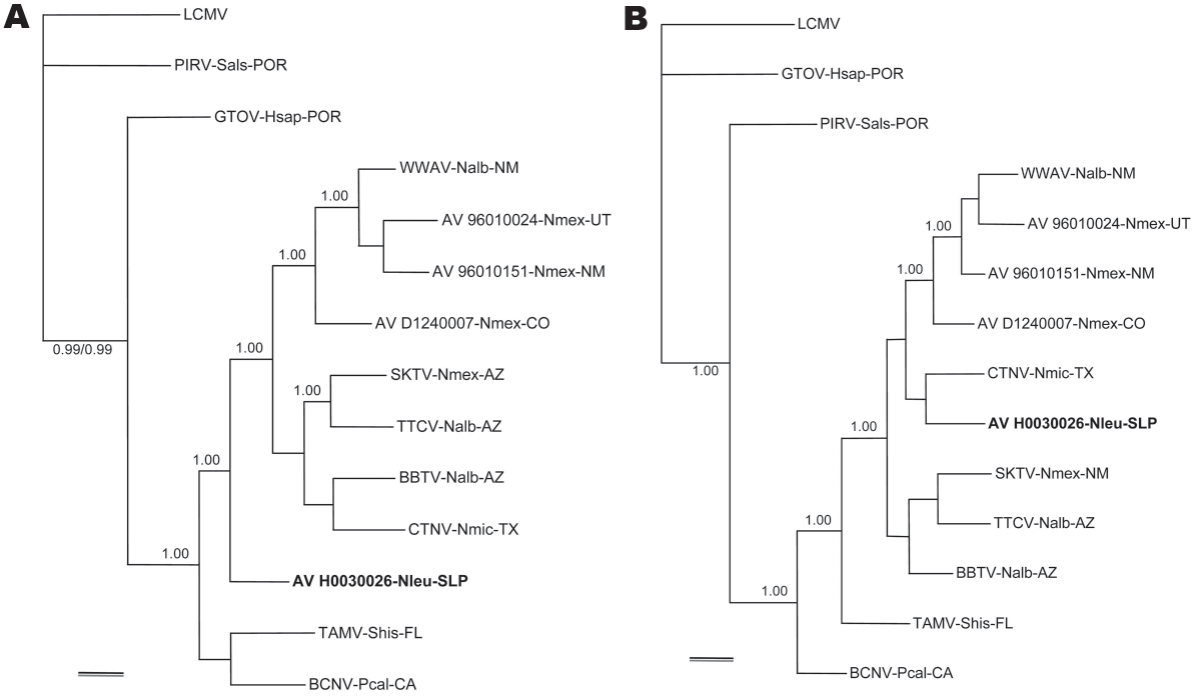
Phylogenetic relationships among 11 North American arenaviruses based on Bayesian analyses of A) glycoprotein precursor gene sequences and B) nucleocapsid protein gene sequences. The number(s) at the nodes are clade probability values, a single 1.00 indicates that the clade probability values for both analyses were 1.00, and clade probability values <0.93 were not included in the phylograms. The branch labels include (in the following order) virus, host species, and state. BBTV, Big Brushy Tank virus strain AV D0390174 (GenBank accession No. EF619035); BCNV, Bear Canyon virus strain AV A0070039 (AY924391); CTNV, Catarina virus strain AV A0400135 (DQ865244); SKTV, Skinner Tank virus strain AV D1000090 (EU123328); TAMV, Tamiami virus strain W 10777 (AF512828); TTCV, Tonto Creek virus strain AV D0150144 (EF619033); WWAV, Whitewater Arroyo virus strain AV 9310135 (AF228063); arenaviruses AV 96010024 (EU123331), AV 96010151 (EU123330), and AV D1240007 (EU123329); GTOV, Guanarito virus strain INH-95551 (AY129247); PIRV, Pirital virus strain VAV-488 (AF485262); LCMV, lymphocytic choriomeningitis virus strain WE (M22138). AZ, Arizona; CA, California; CO, Colorado; FL, Florida; NM, New Mexico; SLP, San Luis Potosí; TX, Texas; UT, Utah; POR, Portuguesa (Venezuela). Nalb, *Neotoma albigula* (white-throated woodrat); Nleu, *N. leucodon* (white-toothed woodrat); Nmex, *N. mexicana* (Mexican woodrat); Nmic, *N. micropus* (southern plains woodrat); Pcal, *Peromyscus californicus* (California mouse); Sals, *Sigmodon alstoni* (Alston’s cotton rat); Shis, *S. hispidus* (hispid cotton rat). Pirital virus and GTOV are South American Tacaribe serocomplex viruses and were selected to represent South American lineages A and B, respectively. The LCMV strain WE is a member of the lymphocytic choriomeningitis-Lassa (Old World) serocomplex and was included in the analyses to enable inference of the ancestral node among the North American arenaviruses. Scale bars indicate 0.1 substitutions per site.

The sequence of the 3,352-nt fragment of the S genomic segment of arenavirus AV H0030026 (GenBank accession no. GQ903697) was determined from RNA isolated from white-toothed woodrat TK133448. The 3,352-nt fragment included the complete glycoprotein precursor (GPC) gene and complete nucleocapsid (N) protein gene of AV H0030026. Nonidentities between the amino acid sequences of the GPC and N protein of AV H0030026 and the amino acid sequences of the GPC and N proteins of the 10 other North American viruses included in the analyses of amino acid sequence data ranged from 28.3% to 35.2% and from 11.6% to 21.0%, respectively (Table 2). The results of the Bayesian analyses of complete GPC gene sequences (Figure 2, panel A) and complete N protein gene sequences (Figure 2, panel B) indicated that AV H0030026 represents a unique phylogenetic lineage but failed to resolve the relationship of AV H0030026 to the other North American viruses analyzed.

**Table 2:** Nonidentities among the predicted amino acid sequences of the glycoprotein precursors and among the predicted amino acid sequences of the nucleocapsid proteins of the North American arenaviruses*

Virus†	% Sequence nonidentity							
	AV H0030026	BBTV	BCNV	CTNV	SKTV	TAMV	TTCV	WWAV
AV H0030026		28.6	35.2	31.3	28.3	33.8	29.2	32.0–33.8
BBTV	12.3		33.8	27.5	26.8	35.4	26.4	30.6–33.7
BCNV	18.0	17.6		34.9	34.0	33.3	34.8	37.4–39.1
CTNV	11.6	10.1	17.8		29.4	36.1	30.0	32.4–34.2
SKTV	13.0	11.0	16.9	11.4		33.3	19.8	30.2–33.3
TAMV	21.0	19.4	21.4	18.5	19.0		35.2	38.0–40.6
TTCV	12.5	11.0	17.1	10.5	9.4	18.9		30.8–33.1
WWAV	13.7–15.5	10.7–13.0	18.3–20.5	12.6–14.6	13.9–15.5	19.2–20.8	13.0–14.1	

*Numbers above and below the diagonal are the nonidentities among the glycoprotein precursors and nucleocapsid proteins, respectively. †BBTV, Big Brushy Tank virus strain AV D0390174 (GenBank accession no. EF619035); BCNV, Bear Canyon virus strain AV A0070039 (AY924391); CTNV, Catarina virus strain AV A0400135 (DQ865244); SKTV, Skinner Tank virus strain AV D1000090 (EU123328); TAMV, Tamiami virus strain W 10777 (AF512828); TTCV, Tonto Creek virus strain AV D0150144 (EF619033); WWAV, Whitewater Arroyo virus strains AV 9310135 (AF228063), AV 96010024 (EU123331), AV 96010151 (EU123330), and AV D1240007 (EU123329). The results of a previous study indicated that AV 96010024, AV 96010151, and AV D1240007 are strains of WWAV or strains of arenaviruses that are phylogenetically closely related to WWAV (*5*). Nonidentities among the amino acid sequences of the glycoprotein precursors and among the amino acid sequences of the nucleocapsid proteins of AV 9310135, AV 96010024, AV 96010151, and AV D1240007 in this study ranged from 16.0% to 25.8% and 7.3% to 10.3%, respectively.

The nucleotide sequence of a 687-nt fragment of the S genomic segment of arenavirus AV H0030028 was determined from RNA isolated from a kidney of white-toothed woodrat TK133451. The sequence of this fragment (GenBank accession no. GU137350) was 99.9% identical to the sequence of the homologous region of the N protein gene AV H0030026. Results of assays for arenavirus RNA in the kidneys of the antibody-positive Nelson’s pocket mice and the antibody-positive kangaroo rat were negative.

## Conclusions

Tacaribe serocomplex viruses have been isolated from white-throated woodrats (*N. albigula*), large-eared woodrats (*N. macrotis*), Mexican woodrats (*N. mexicana*), southern plains woodrats (*N. micropus*), and a bushy-tailed woodrat (*N. cinerea*) captured in the western United States (*2*–*5*,*7*,*8*), and antibody to Whitewater Arroyo virus has been found in woodrats captured in San Luis Potosí (M.L. Milazzo, unpub. data). Our results indicate that an arenavirus is naturally associated with the white-toothed woodrat (*N. leucodon*) and that Tacaribe serocomplex viruses are enzootic to Mexico.

The results of the Bayesian analyses indicated that AV H0030026 is phylogenetically distinct from other North American arenaviruses. Sequence nonidentities between the GPC of AV H0030026 and the GPC of the other North America arenaviruses (Table 2) were substantively greater than sequence nonidentities between the GPC of strains of phylogenetically closely related South American arenavirus species (*3*). Furthermore, sequence nonidentities between the N protein of AV H0030026 and the N proteins of the other North American arenaviruses (Table 2) were comparable to the amino acid sequence nonidentities between the N proteins of strains of phylogenetically closely related South American arenavirus species (*3*). Thus, AV H0030026 may represent a novel species (proposed name Real de Catorce) in the family *Arenaviridae*, genus *Arenavirus* (*9*).

Nelson’s pocket mouse (*C. nelsoni*) and Merriam’s kangaroo rat (*D. merriami*) are members of the rodent family Heteromyidae. No accounts of arenavirus infections in these or other heteromyid rodents have been published. The anti-arenavirus antibody in the pocket mice and kangaroo rat in this study may be a consequence of infection with an arenavirus other than Real de Catorce virus. Alternatively, the results of the ELISA on these rodents are inaccurate (i.e., falsely positive).

Five Tacaribe serocomplex viruses are known to naturally cause severe febrile disease in humans: GTOV, Junín, Machupo, Sabiá, and Chapare viruses. The diseases caused by these viruses range from sporadic cases to small outbreaks to hyperendemic episodes. Humans usually become infected with arenaviruses by inhalation of virus in aerosolized droplets of secretions or excretions from infected rodents. Other means of infection may include ingestion of infected rodents (*13*).

Human consumption of woodrats is common in rural regions in the highlands of Mexico. For example, white-throated woodrats are consumed by humans in the Potosí-Zacatecan Mexican Plateau (*14*), and Mexican woodrats (*N. mexicana*), Mexican deer mice (*Peromyscus mexicanus*), and other cricetid rodents are consumed by the Tzeltzal Indians in Chiapas (*15*). Future studies on arenaviruses native to North America should include assessments of whether humans who consume woodrats or live or work in close association with woodrats are infected by the Tacaribe serocomplex viruses associated with these rodents.
